# P119: Hand hygiene compliance among nurses in a Japanese tertiary hospital emergency department

**DOI:** 10.1186/2047-2994-2-S1-P119

**Published:** 2013-06-20

**Authors:** S Ikeda, K Tokuda, H Kanamori, Y Hirai, S Endo, H Kunishima, M Kaku

**Affiliations:** 1 Infection Control Unit, Tohoku University Hospital, Sendai, Japan; 2 Department of Infection Control and Laboratory Diagnostics, Internal Medicine, Tohoku University Graduate School of Medicine, Sendai, Japan

## Introduction

In June 2012, the number of patients in whom MRSA was detected increased in our hospital emergency department. As hospital-acquired infection was suspected in some of the patients, we conducted observation-based evaluation of hand hygiene practices.

## Objectives

To evaluate the status of hand hygiene compliance in nurses working at the emergency department.

## Methods

We conducted a total of 11 surveys between June and August 2012 on the status of hand hygiene compliance in 60 nurses at the emergency department. Two infection control practitioners investigated the five moments requiring hand hygiene. The survey form developed by WHO was used. For statistical analysis, chi-square test was conducted using Epi Info™ 3.5.4.

## Results

A total of 435 moments requiring hand hygiene were observed, including 64 moments before touching a patient (Moment 1), 112 moments before a clean/asceptic procedure (Moment 2), 40 moments after body fluid exposure (Moment 3), 99 moments after touching a patient (Moment 4), and 120 moments after touching patient surroundings (Moment 5). The median of overall compliance rates was 38% (range, 21-69%). The compliance rate was the highest for Moment 3 at 52.5%, followed by Moment 4 at 47.5%, Moment 5 at 35.0%, Moment 2 at 33.9%, and Moment 1 at 33.9%. With regard to Moments 2 to 4, the compliance rates in nurses wearing gloves were significantly lower than those not wearing gloves.

## Conclusion

Our data showed that the overall compliance rate in the emergency department was low as previously reported in other countries. However, we found that there were differences in compliance rates by moment, and the compliance rates were particularly low before touching a patient and before a clean/asceptic procedure. Therefore, education targeting moments with low compliance rates will be required. As the compliance rate was unexpectedly low when gloves were worn, re-education concerning appropriate use of gloves is considered to be necessary.

## Disclosure of interest

None declared

## References

[B1] Erasmus V Daha TJ Brug H Richardus JH Behrendt MD Vos MC van Beeck EF Systematic review of studies on compliance with hand hygiene guidelines in hospital care Infect Control Hosp Epidemiol 2010 31 3 283 94 10.1086/650451 20088678

